# Draft Genome Sequences of Seven Multidrug-Resistant *Acinetobacter baumannii* Strains, Isolated from Respiratory Samples in Spain

**DOI:** 10.1128/genomeA.00083-16

**Published:** 2016-03-31

**Authors:** Gema Labrador-Herrera, Rocío Álvarez, Rafael López-Rojas, Younes Smani, Tania Cebrero-Cangueiro, Antonio Rueda, Javier Pérez Florido, Jerónimo Pachón, María Eugenia Pachón-Ibáñez

**Affiliations:** a Unit of Infectious Diseases, Microbiology and Preventive Medicine and Biomedical Institute of Seville (IBiS), University Hospital Virgen del Rocio/CSIC/University of Seville, Seville, Spain; b Infectious Diseases and Clinical Microbiology Unit, University Hospital Virgen Macarena, Seville, Spain; c Medical Genome Project, Genomics and Bioinformatics Platform of Andalusia (GBPA), Seville, Spain

## Abstract

The draft genome sequences of seven multidrug-resistant *Acinetobacter baumannii* clinical strains belonging to sequence types ST-208 and ST-218 are reported in this study. They were isolated from tracheobronchial aspirate of mechanically ventilated adult patients admitted to the intensive care unit of a Spanish tertiary hospital during 2010 to 2011.

## GENOME ANNOUNCEMENT

*Acinetobacter baumannii* is among the leading etiologies of hospital-acquired infections, particularly in critically ill patients (1). The increasing prevalence of multidrug-resistance reduces available treatments and threatens public health (2). During 2010 to 2011, 207 multidrug-resistant (MDR) *A. baumannii* strains were isolated from 100 adult patients in the intensive care unit of a tertiary hospital in Seville, Spain.

In this study, we present the draft genome sequences of 7 of these MDR *A. baumannii* clinical strains (including resistance to gentamicin, imipenem, meropenem, ceftazidime, cefepime, ticarcillin, trimethoprim-sulfamethoxazole, tetracycline, levofloxacin, and ciprofloxacin; and susceptibility to colistin) isolated from tracheobronchial aspirates. Whole-genome sequencing was performed using the Illumina MiSeq platform with 300 bp paired-end reads, resulting in mean genome coverage of 25.2 ± 5.5-fold (mean ± SD of the 7 strains). Reads were preprocessed with Trimmomatic v0.32 (http://www.usadellab.org/cms/?page=trimmomatic) (3) and *de novo* assembled with ABySS assembler v1.5.2 (http://www.bcgsc.ca/platform/bioinfo/software/abyss) (4), resulting in 381 ± 52 contigs. The assemblies were filtered for contigs larger than 500 bp, leaving 210 ± 34 contigs with a total length of 4,036,456 ± 123,611 bp and an *N*_50_ of 47,317 ± 8,993 bp. The G+C content was 39.2 ± 0.1%. Gene prediction and annotation were performed utilizing GeneMark v2.5 (http://exon.gatech.edu/GeneMark/) and RAST server v1.0 (http://rast.nmpdr.org) (5), respectively, yielding a total of 3,876 ± 134 coding sequences and 70 ± 15 RNAs. Two different sequence types (ST) were found with MLST v1.8 using the Oxford database (https://cge.cbs.dtu.dk/services/MLST/) (6, 7). Most of the strains belong to ST-208, except AbMDR-GLH5 and AbMDR-GLH6 that belong to ST-218. With pulsed-field gel electrophoresis, 3 pulsotypes were found: pulsotype 1 (AbMDR-GLH2 and AbMDR-GLH4), pulsotype 2 (AbMDR-GLH1, AbMDR-GLH3, and AbMDR-GLH8), and pulsotype 3 (AbMDR-GLH5 and AbMDR-GLH6). To identify genes conferring antimicrobial resistance, the bioinformatic tool ResFinder v2.1 was used (https://cge.cbs.dtu.dk/services/ResFinder/) (8). The aminoglycoside resistance genes *aac(6′)-II*, *strB*, and *strA*, were detected in pulsotype 1 strains, while pulsotype 2 strains carry *aac(3)-Ia*, *aadA1*, *strB*, and *strA* genes, and pulsotype 3 strains carry *aac(6′)-II*, *aac(3)-IIa*, *strB*, and *strA* genes. Resistance genes *blaOXA-58*, *blaOXA-66*, and *blaADC-25*, which confer β-lactam resistance, were found in strains from pulsotypes 1 and 2; nevertheless, pulsotype 3 strains carry *blaOXA-109* and *blaADC-25* genes. Referring to sulfonamide resistance, all the strains have *sul1* gene, and strains from pulsotypes 1 and 2 also possess *sul2* gene. The gene *tet(B)*, related with tetracycline resistance, was found in all the strains. Furthermore, point mutations in genes associated with quinolone resistance were found in all the strains using BLAST (Basic Local Alignment Search Tool) (http://blast.ncbi.nlm.nih.gov/Blast.cgi) (9) comparing with genes from susceptible strains.

### Nucleotide sequence accession numbers.

These whole-genome shotgun (WGS) projects have been deposited at DDBJ/EMBL/GenBank under the accession numbers LIWE00000000 (AbMDR-GLH1), LIZZ00000000 (AbMDR-GLH2), LJAA00000000 (AbMDR-GLH3), LJAB00000000 (AbMDR-GLH4), LJAC00000000 (AbMDR-GLH5), LJAD00000000 (AbMDR-GLH6), and LJAF00000000 (AbMDR-GLH8). The versions described in this report are the first versions, LIWE01000000, LIZZ01000000, LJAA01000000, LJAB01000000, LJAC01000000, LJAD01000000, and LJAF01000000.
